# Onwards and upwards: towards a sustainable future

**DOI:** 10.1098/rsos.172294

**Published:** 2018-01-24

**Authors:** Jeremy K. M. Sanders

**Affiliations:** Department of Chemistry, University of Cambridge, Lensfield Road, Cambridge CB2 1EW, UK

*Royal Society Open Science* celebrated its third birthday in September 2017, and as we look ahead to fresh successes, the start of 2018 seems an opportune moment to pick out some journal highlights from the last 12 months:

— We received over 2067 submissions, resulting in more than 640 accepted papers.
— The journal received and published its first Computer Sciences Registered Report, which explored ‘…the relationship of university performance and its global physical connectedness' [1] and generated wide interest.
— Working with the Alan Turing Institute, who kindly hosted the journal, we launched our first completed special article collection in February 2017, examining the multidisciplinary topic of ‘City Analytics' (https://www.youtube.com/watch?v=OQVMpHRojdw (accessed 20 December 2017)). All the papers in the collection are available at http://royalsocietypublishing.org/cc/city-analytics and make for fascinating reading.
— Our collaboration with the Royal Society of Chemistry goes from strength to strength, with 20% of submissions now being received from this route. The success of the collaboration shows the value and importance of joint work by learned Societies in promoting high-quality science regardless of impact.
— We have encouraged Royal Society University Research Fellows and Dorothy Hodgkin Fellowship holders initially in the materials and chemical sciences to submit their work to the journal. Recently published examples include Dr Asel Sartbaeva and co-workers [2], and Dr Benjamin Morgan [3], but keep an eye on your eToCs for more papers in this ‘Next Generation' collection very soon.

Beyond the undoubted scientific success of *Royal Society Open Science*, it has been gratifying to see the wide coverage the journal regularly receives in the popular press. A couple of noteworthy examples spring to mind: firstly, at 17 years old, possibly our youngest author published ‘The Electric Honeycomb; an investigation of the Rose window instability' [4]. This paper, and its accompanying blog (the video is worth watching) (https://blogs.royalsociety.org/publishing/electric-dreams/ (accessed 20 December 2017)), is a fine example of *Royal Society Open Science* supporting research regardless of its source. More recently, it transpires that sheep are surprisingly good at recognizing human faces (whether film stars or former American Presidents) [5].

With these firm foundations in place, it is now time to look to the future for *Royal Society Open Science*. To this end, readers may be aware that from 1 January 2018 all new submissions will be subject to a modest article processing charge if accepted for publication (https://blogs.royalsociety.org/publishing/building-a-sustainable-open-science-journal/ (accessed 20 December 2017)). This will ensure that the journal, which has been supported by the Royal Society during its launch phase, will remain sustainable and continue to drive publishing innovation at the Society. The charge will cover the costs of maintaining a dedicated in-house editorial office, managing the peer-review process, providing copyediting and typesetting services, publication and hosting services, as well as enabling the Royal Society to effectively promote authors' work. In the event that a small surplus is generated, this will be used by the Royal Society to support its range of activities promoting and supporting high-quality science and public engagement with the work it undertakes. Nevertheless, the journal recognizes that even modest charges may be unaffordable for some authors, and this is why we have prepared a generous waiver policy (http://rsos.royalsocietypublishing.org/article-processing-charge-waivers (accessed 20 December 2017)). In summary, the waiver will apply to any author who does not have the ability to pay for publication, though the journal does reserve the right to ask authors for evidence to support the waiver application. With the waiver in place, we will not be disadvantaging our geographically diverse authorship.

A number of our Associate Editors completed the end of their term of service at the end of 2017, and we wish them all the best in their future endeavours. The early successes of the journal would not have been possible without their hard work. We welcome the newly joining members of the Associate Editorial Board, and look forward to working with them to develop the journal. The next year will see *Royal Society Open Science* exploring ways to recognize the support we receive from a small army of volunteer referees—we remain very grateful to them for their assistance. Indeed, we will shortly publish our latest list of referees who opted to have their work for the journal recognized.

We hope you will join us on the next steps of our journey, submitting your work to the journal, refereeing when asked, and reading and sharing the manuscripts published by *Royal Society Open Science*.

## Data accessibility

Data reported are supplied to the Editors and can be requested from the Editorial office.
